# The Assessment and Comparison of the Knowledge of Breast Self-Examination and Breast Carcinoma Among Health Care Workers and the General Population in an Urban Setting

**DOI:** 10.7759/cureus.36592

**Published:** 2023-03-23

**Authors:** Shahveer Polishwala, Shagun ., Shilpa Patankar

**Affiliations:** 1 Department of Surgery, Bharati Hospital and Research Center, Pune, IND; 2 Department of Medicine, Buddhist Mission Hospital, Ranchi, IND

**Keywords:** womens health care, breast health, breast malignancy, breast cancer prevention, breast tumour, breast cancer, breast self-examination

## Abstract

Background

Breast cancer is one of the common causes of cancer related mortality in women. Early detection and treatment can combat the morbidity and mortality of breast cancer. Most first-world countries have a screening program to facilitate early detection of breast malignancy. A lack of similar programs in developing countries, compounded with ignorance and financial crunch, often leaves women vulnerable due to late detection and complications. Identification of early physical changes in breasts through regular breast self-examination (BSE) can potentially aid in the early detection of breast lumps. Ideally, all women should have access to screening programs, although, practically, it is difficult to achieve mass screening in resource-poor areas. BSE cannot completely bridge this gap in health care; however, it can undoubtedly aid in increased awareness, identification of danger signs, and timely approach to the health care center for intervention.

Materials and method

A cross-sectional study was conducted at Bharati Vidyapeeth Medical College, Pune, India. The participants were administered a pretested questionnaire to collect information about their understanding of BSE. The data were analyzed using the Statistical Package for Social Sciences (SPSS) statistical software, Version 25. Mean and frequencies were used to compare participants from various backgrounds.

Results

The total sample consisted of 1,649 women from various educational backgrounds. Every doctor had heard about BSE compared to 81% of women from the general population; 84% of doctors and less than 40% of women representing the general population were taught to perform BSE; however, only around 34% of all women perform BSE. Women from the general population were largely unaware of the correct age to begin BSE, the frequency of performance, its correlation with the menstrual cycle, and the steps necessary to perform it. Women employed in the health care industry were better informed than the general population but still needed to be aware of BSE's details.

Conclusion

The study highlighted the lack of information regarding breast malignancy and self-examination among women from all educational and professional backgrounds. Women in the health care sector are better informed about the topic than the general population but still lack adequate information. There is a dire need to train women about the procedure, frequency, and correct time of conducting BSE and the telltale signs of breast carcinoma. Women in the health care industry can be educated and trained as educators on the topic, who can further disseminate the information to the general population to promote early detection of breast malignancy.

## Introduction

Annually, 18.1 million new cancer cases are registered across the globe, and 9.6 million people are lost to cancer-related deaths; 11.6% of all registered cancer cases are of breast cancer, ranking it as the second most common cancer affecting women globally. Breast cancer has a significant mortality potential and is responsible for most cancer-related deaths globally [1].

Breast cancer, although it affects a large population, is mostly treatable with early diagnosis and hence commands an immediate need for breast cancer education and intervention strategies [2]. Screening programs are in place in developed countries, with a high prevalence of breast cancer for its early detection, assisted by the far-reaching health care systems. The importance of such screening programs in developing countries, with lower incidences and prevalence of breast malignancy, is still unclear. Although the incidence of breast cancer in developing countries is relatively low, women tend to be diagnosed mostly with advanced stage, leading to higher mortality rates than those in the developed nations [3].

Preventive action implementation has been recognized as the primary element in the battle against breast cancer on a global scale. The globally recommended screening procedures for the early identification of breast cancer include breast self-examination (BSE), clinical breast examination (CBE), and radiological imaging such as mammography. Screening breast cancer by mammography is the established gold standard unachievable for many countries at the moment. For this screening tool to achieve its described potential, its regularity should be maintained by a national public health policy, or the nation’s per capita income should be high enough to bear the cost. In developing nations and countries needing a national health scheme, mammograms are not a norm due to high economic liability, difficulty in follow-up, and unsatisfactory medical adherence, which results in a high proportion of cases diagnosed at an advanced stage [4].

BSE is an easy, patient-centered, private, inexpensive, non-invasive, reliable, and effective tool that requires only hands and the eyes. This tool is readily available to all women from all socioeconomic realities at all times in the world and takes only five minutes to perform [5-7]. Some studies emphasize that BSE has a relative early detection potential for properly trained women familiar with the structure and changes in their breasts and hence can be the first to discover breast lumps [6,8,9].

It is noteworthy that studies worldwide have found that a high proportion of breast changes are detected by women and their partners who raise the alarm for cancer diagnosis. The literature claims that approximately two-thirds of women [10] of low-income strata detect their cancers, and the range can go as high as 80% [5] to 84.6% [8].

A national survey conducted by the American College of Surgeons (ACS) in the early 1980s noted that 73% of breast tumors were found by patients [10]. Although this study dates back to the time before mammography screening was widely available, it still emphasizes the importance of BSE in areas where regular mammography screening is still not a norm.

Keeping in mind the health and safety of women from economically, racially, and technologically underprivileged areas of the world, the “Breast Health Global Initiative” (BHGI) encourages BSE at the basic level [4]. Organizations such as the National Cancer Institute and the American Cancer Society (ACS) have also previously recommended BSE [7]. In 2003, ACS revised guidelines on BSE and removed it as a mandatory screening tool. ACS, however, emphasizes that women should be educated about the advantages and limitations of BSE [9]. As per the guidelines by ACS, women should be aware of their breast structure and report to a physician if something changes [6]. Certain cohort studies, case-control studies, and case-series studies have indicated that mortality decreased in women who performed high-quality BSE [9].

For the past few decades, researchers have questioned the utility and reliability of BSE, citing the incidence of false-positive lumps leading to the wastage of resources on the further workup of these lumps and emotional harm to the woman.

One of the most prominent studies advocating against BSE was conducted in Shanghai over 30 years ago in a population that is not very genetically predisposed to breast cancer for only five years and included already prevalent cases. Given the short follow-up period and the lower rate of breast cancer in this geographical area, the study gathered information about increased workups and false-positive results. However, it failed to provide reliable data on mortality benefits. The data on mortality benefits were unreliable since BSE does not play a role in decreasing mortality in prevalent cases [7].

While the negative emotional effect of BSE and false-positive cases have raised questions, some researchers have pointed out that the fault lies in implementing BSE and not the concept in general. Earlier, women were taught to detect multiple small lumps, usually benign, instead of larger hard lumps, which turn out to be malignant [5]. The implementation can be corrected with proper dissemination of correct information to decrease false-positive cases significantly.

The most important aspect of harvesting the potential reduction in mortality through BSE is to empower women to competently and correctly perform it and to ensure the availability of accessible diagnostic follow-up if needed [6,11]. BSE should not be abandoned as a screening tool as it motivates women to seek medical attention [7]. It is associated with the discovery of otherwise undetectable lumps [9] and smaller sizes of the tumor at diagnosis [5,11]. The need of the hour is not to scrape off this tool but to allow women to manage their health by educating them about the procedure and discussing its limitations. BSE should be an add-on to regular mammography in resource-positive areas and not a replacement [9].

While women need to be “breast aware,” it is often hampered by the lack of knowledge regarding BSE in the general population [12]. Studies have highlighted that the percentage of women performing BSE is low, even among doctors, which is around 21% [13].

With this background, the present study is designed to establish the baseline awareness regarding BSE by assessing the knowledge, behavior, and practices surrounding it among female health care workers and the general population. The authors hypothesize that there will be a significant difference between the knowledge levels of health care workers and the general population, with a higher than satisfactory level of information among health care workers.

## Materials and methods

From March 2022 to April 2022, a cross-sectional, randomized, observational study was conducted at Bharati Vidyapeeth Medical College (BVDUMC), Pune. The institutional ethical committee of BVDUMC approved this study in October 2021 with the reference number BVDUMC/IEC/14. A total of 1,700 women were interviewed for this study, of which only 1,649 completed forms were considered. All participants were requested to sign an informed electronic consent.

The inclusion criteria for the study were any women aged 18 to 65 within the health care premises of Bharati Hospital and Research Centre, Pune, consenting to participate. The study included doctors, nurses, and medical students to represent awareness among health care workers. For the representation of the general population, hospital workers not directly involved in patient care, referred to as other hospital workers, female relatives, and visitors of patients coming to Bharati Hospital's OPDs and wards, were interviewed.

Women already diagnosed with breast cancer were excluded. To avoid bias, doctors with post-graduate degrees in general surgery and final-year medical students were excluded from the study.

Data were collected through a structured, self-administered Google form containing a questionnaire prepared after a review of the literature. Questions on breast cancer and BSE, including practical questions about the method of performing BSE, were formed in a multiple-choice format. The questionnaire was pretested on 39 volunteers from the medical college. The questionnaire has been compiled and included in the appendices. All the participants were redirected to a link leading to a YouTube video explaining BSE after the survey.

Data were collected with the help of volunteers trained to interview each participant personally and help them technically while filling out the Google form. The vernacular language was used to explain each question to the participants to ensure an adequate response rate. No coercion was used to obtain the data.

The collected data were tabulated in Excel and were cleaned and cross-checked to remove any incomplete forms. Statistical Package for Social Sciences (SPSS) statistical software Version 25 (IBM Corp., Armonk, NY) was used to analyze the data. Tables and text were used to present frequencies and percentages, and mean and standard deviation were used for descriptive statistics. The study used a p-value of <0.5 to declare a significant association between the knowledge of BSE and breast carcinoma among other hospital workers, medical students, doctors, and the general population in an urban setting.

## Results

Sociodemographic characteristics

The study took into account 1,700 respondents. A sample size of 1,649 was achieved, giving us a response rate of 97% after excluding 51 incomplete forms. Women aged 18 to 65 years were interviewed, and the sample had a mean age of 32 years (SD ± 11.8). While 57.1% of the respondents were married, 41% had never been married. Participants from various strata of educational background were included, including but not restricted to medical students (11.2%), doctors (7.8%), and nurses (7.9%). More participants consumed non-vegetarian food (62.4%) than vegetarian food. Most participants followed Hinduism (89.9%) as their religious belief. The details of the demography are depicted in Table 1.

**Table 1:** Frequency distribution of demographic distributions

		Frequency (n)	Percentage (%)
Marital status	Married	942	57.1
	Single/never married	680	41.2
	Separated/divorced/widowed	27	1.6
Religion	Hindu	1482	89.9
	Muslim	64	3.9
	Jain	30	1.8
	Christian	24	1.5
	Others	49	2.9
Occupation	General population	1094	66.3
	Doctors	129	7.8
	Nurses	130	7.9
	Other hospital workers	111	6.7
	Medical students	185	11.2
Diet	Non-vegetarian	1029	62.4
	Vegetarian	620	37.6

Knowledge about breast cancer and its prevention

Of all participants, 85.8% had ever heard of breast cancer, with the highest prevalence among doctors (100%) and medical students (100%), as shown in Table 2. Only 81.62% of women in the general population had heard about breast cancer. Around 14.5% of all participants had a family member diagnosed with breast cancer. The participants were asked to select the best options from a list of probable risk factors to understand their knowledge better. A total of 47.6% of participants identified increasing age and family history as risk factors. Around 41% of patients thought obesity, alcohol, and smoking posed a risk. A large percentage of doctors identified increasing age and family history (93.7%), and obesity, alcohol, and smoking (66%) as risk factors. A similar reading was obtained from the survey of medical students. At the same time, the interview of nurses highlighted that a significantly lesser number of them considered alcohol, obesity, and smoking carcinogenic. All-inclusive data collected from participants reflected that they were unaware of various signs of breast cancer. While some characteristics such as lumps in the breast and armpits were well-recognized by all, others such as discharge from the nipple and retraction of nipples, skin changes, and weight loss were not seen as danger signs. A very significant difference in opinion was seen between doctors and the general population about skin changes, nipple discharge, and retraction of nipples as signs of cancer. The general population and other hospital workers were largely ignorant of these symptoms of breast cancer.

**Table 2:** Association between risk factors and occupation

		Occupation category					Total (n=1,649)	Chi-square value	p-value
		Doctors (n=129)	Other hospital workers (n=111)	Medical students (n=185)	Nurses (n=130)	Others (n=1094)			
Have you heard about breast cancer?	No	0	27	0	6	201	234	86.79	<0.001
	Yes	129	84	185	124	893	1,415		
Has any member of your family been diagnosed with breast cancer?	No	104	105	157	128	915	1,409	30.52	<0.001
	Yes	25	6	28	2	179	240		
Is increasing age/family history a risk factor?	No	8	61	56	41	698	864	226.71	<0.001
	Yes	121	50	129	89	396	785		
Is obesity/alcohol/smoking a risk factor?	No	43	74	72	83	701	973	81.59	<0.001
	Yes	86	37	113	47	393	676		
Is poor sleep/heavy lifting a risk factor?	No	114	107	157	126	994	1,498	18.79	0.001
	Yes	15	4	28	4	100	151		
Is lump in the breast/armpit a sign of breast cancer?	No	6	14	9	17	155	201	20.24	<0.001
	Yes	123	97	176	113	939	1,448		
Is discharge from the nipple/retraction of the nipple a sign of breast cancer?	No	13	73	49	61	691	887	202.19	<0.001
	Yes	116	38	136	69	403	762		
Are skin changes in the breast a sign of breast cancer?	No	17	84	63	74	777	1,015	238.19	<0.001
	Yes	112	27	122	56	317	634		
Is drastic loss of weight a sign of breast cancer?	No	43	102	139	119	964	1,367	265.67	<0.001
	Yes	86	9	46	11	130	282		

The questionnaire asked the participants to select their source of information about breast cancer. As shown in Table 3, media sources play the most crucial role in dispersing information about breast cancer among the interviewed women. A significant percentage of women get their information from doctors and nurses in the hospital setting. Friends, family members, and teachers contribute the least to the dissemination of information.

**Table 3:** Different sources of obtaining information on breast cancer

	Frequency (n)	Percentage (%)
Media (TV, radio, internet, etc.)	949	57.6
Hospitals/doctors/nurses	674	40.9
Books	533	32.3
Lectures/conferences/seminars	410	24.9
Friends/family members/teachers	102	6.2

Knowledge about application and practice of BSE

The questionnaire mainly dealt in depth with the practices of BSE. Women were questioned if they were aware of the practice of BSE and if those who had heard about it practiced it. They were asked about the ideal age of initiation, frequency of practice, and the best time to perform it. From the total sample of 1,649, around 85% of respondents had heard about BSE, with almost all doctors, medical students, and nurses being aware of it. Nearly 50% of other hospital workers and the general population also knew it about it. Only 37.5% of women had been taught how to perform it, with the highest positive response from doctors and nurses. Half of the medical students had been taught to conduct a BSE, whereas less than 37% of women from the general population had received instructions; 30.4% of participants had yet to learn the age at which BSE should be started, whereas 42% believed it should be started at 19 years. Only 40% of women selected a monthly frequency to perform BSE, whereas 34% needed to be more knowledgeable about the recommended examination frequency. More than 50% of the respondents needed to learn the correlation between the menstrual cycle and the best time to perform BSE. As Table 4 depicts, 1,089 participants out of 1,649 denied practicing BSE. The difference in correct knowledge about BSE was significant when a comparison was drawn between doctors, medical students, nurses, and the general population (p<0.001).

**Table 4:** Basic knowledge about BSE BSE, breast self-examination

		Occupation					Total (n=1,649)	Chi-square value	p-value	
		Doctors (n=129)	Other hospital workers (n=111)	Medical students (n=185)	Nurses (n=130)	Others (n=1049				
										Have you ever heard of BSE?	No	2	57	25	16	480	580	180.91	<0.001	
Yes	127	54	160	114	614	1,060													
Do you practice BSE?	No	36	74	121	65	793	1,089	118.87	<0.001	
	Yes	93	37	64	65	301	560			
Have you been taught how to do BSE?	No	20	72	102	36	799	1,029	244.85	<0.001	
	Yes	109	39	83	94	295	620			
At what age should BSE be started?	After menopause	3	5	1	1	44	54	182.48	<0.001	
	From 30 years of age	50	26	27	26	251	380			
	From birth	0	1	0	2	8	11			
	From 20 years	75	38	131	78	380	702			
	No idea	1	41	26	23	411	502			
How often should BSE be performed?	Daily	3	6	5	8	42	64	114.25	<0.001	
	Monthly	83	30	98	61	388	660			
	No idea	4	49	50	30	431	564			
	Weekly	25	15	21	26	133	220			
	Yearly	14	11	11	5	100	141			
What is the best time to do BSE?	A week after period	94	22	70	53	280	519	182.08	<0.001	
	During breastfeeding	0	7	3	5	44	59			
	During menstrual flow	10	16	12	13	67	118			
	During pregnancy	0	4	4	14	43	65			
	No idea	25	62	96	45	660	888			

When asked about the steps included in BSE, women from the general population primarily selected palpation of the breast and armpits with the hands and inspection of breasts in the mirror, as depicted in Table 5. They were aware that no radiological process was involved in BSE, and almost all of them excluded ultrasonography and mammography from BSE.

**Table 5:** Methods to perform BSE and modes of action after the discovery of lumps BSE, breast self-examination

		Occupation category					Total (n=1,649)	Chi-square value	p-value
		Doctors (n=129)	Other hospital workers (n=111)	Medical students (n=185)	Nurses (n=130)	Others (n=1094)			
Breast BSE is done by doing an ultrasound of the breast	No	126	111	185	130	1094	1,646	35.41	<0.001
	Yes	3	0	0	0	0	3		
BSE is done by inspecting the breast in the mirror	No	45	23	80	35	243	426	43.86	<0.001
	Yes	84	88	105	95	851	1,223		
BSE is done by feeling the breast/armpit with the hand	No	6	31	20	32	316	405	58.19	<0.001
	Yes	123	80	165	98	778	1,244		
BSE is done by mammography	No	115	86	145	98	848	1,292	10.04	0.04
	Yes	14	25	40	32	246	357		
If you discover any abnormality during BSE, what will you do?	Do nothing	0	1	2	0	19	22	8.42	0.75
	Do some lab tests	4	6	8	7	40	65		
	Pray over it	0	0	1	1	5	7		
	See a doctor	125	104	174	122	1030	1,555		

The final section considers attitudes toward BSE as a practice and its benefits. The majority of women from all backgrounds of education knew that BSE helped in the early detection of abnormal changes in breast and breast cancer, and it is an excellent practice to indulge in. The details are mentioned in Table 6.

**Table 6:** BSE as a practice BSE, breast self-examination

		Occupation category					Total (n=1,649)	Chi-square value	p-value
		Doctors n=129	Other hospital workers (n=111)	Medical students (n=185)	Nurses (n=130)	Others (n=1094)			
What are the benefits of BSE?	A good breast exercise	0	6	3	4	63	76	50.73	<0.001
	Detection of any abnormal changes in the breast	49	25	67	22	264	427		
	Early detection of breast cancer	80	75	112	102	714	1,083		
	No benefits	0	5	3	2	53	63		
Do you think BSE is a good practice?	No	2	5	3	4	63	77	10.34	0.04
	Yes	127	106	182	126	1,031	1,572		

## Discussion

Breast cancer is one of the most common cancers, affecting 11.6% of the global population and resulting in millions of fatalities annually [1]. Early detection and timely intervention can significantly reduce morbidity and mortality associated with breast cancer.

The central health care programs of every country are tailored to meet the health and economic needs of the population. There are population-wide breast screening programs in high-income countries like the United States and Australia, which encourage women over the age of 50 years to have mammograms every 2 years [14]; such universal screening schedules are not the most feasible option for a large population of resource-poor areas citing the dearth of clinical check-up facilities, radiological centers, awareness, and the prevalence of taboos among women.

 Educating women about the signs and symptoms of breast cancer and encouraging them to reach out to clinical centers in case of any breast changes can help in early detection. BSE is a handy, accessible, and "zero-cost" tool for women from resource-deprived areas to screen for any breast changes [5-7].

This study includes women from almost all walks of the population. Women falling in the broad age bracket of 18 to 65 years were surveyed to encompass the reproductive age group and the at-risk population. Data were collected from women from medical and non-medical backgrounds to highlight and establish the baseline level of information and alertness, which can help government and local agencies to formulate their educational programs.

The study results show a stark difference in the basic information and practices about breast cancer prevention and BSE among women from medical and non-medical backgrounds in India. As hypothesized, awareness regarding breast malignancy and the practice of BSE is higher among doctors, nurses, and medical students; women from non-medical backgrounds are comparatively unaware. It is, however, noticed that contrary to the hypothesis, the awareness among health care workers, although higher, is still not satisfactory.

The study highlighted an optimum level of information about the general awareness of breast cancer, with an aggregate of the total population reaching 85%, comparable to 84% of awareness noted in a meta-analysis [15]. Women not employed in health care were largely unaware of breast cancer risk factors and symptoms such as skin changes, nipple discharge, retraction of nipples, and weight loss. This was in contrast with the data from a meta-analysis that reported a higher awareness level [15]. The most widely recognized risk factor was a family history of breast cancer, and the most identified symptom was a breast lump. Women largely credited media as the most crucial source of breast cancer information, as has been reported in another study [16]. These women are mainly unaware of BSE, with awareness statistics significantly lower than studies conducted for students in Sharjah, UAE [16], Ethiopia [17], and Saudi Arabia [18], but higher than the level of awareness seen in another study conducted in Ethiopia [19].

Our study showed a vast difference in the training regarding BSE in different strata of society, as was expected. Less than 40% of women representing the general population were taught to perform BSE. Only 28% of these women admitted to practicing BSE. This is higher than the BSE practiced by 18.5% of women in Turkey and lower than 42% of women in Saudi Arabia [18]. Another meta-analysis differentiated between “ever and regular” BSE practice and found the prevalence to be 44% and 17.9%, respectively [20].

The correct age to begin BSE was misidentified by women from all backgrounds, including doctors. The overall percentage was still higher than the study conducted in Ethiopia, where the aggregate was 32% [17]. There is a need to educate women about the correct age for commencing BSE, including the health care staff, who seemed uncertain about it. Women are largely unsure about the frequency of performing BSE, as was found in other studies [17,19,21].

Our study pinpointed the lack of basic understanding and awareness about the steps of BSE in the whole sampled population. Most women could differentiate between BSE and radiological examinations such as ultrasonography and mammography and knew that BSE did not include them as a part of it.

Another contribution of our study is establishing that health care workers need to be educated about BSE. Doctors, nurses, and medical students were better informed when compared to the general population, but the level of understanding was unsatisfactory. The caregivers were not appropriately equipped to answer the queries of the masses or educate them about the topic.

It was noted that although the study subjects did not have adequate knowledge and were negligent about the exact procedure of performing BSE, they were optimistic about it. They believed it to be a good screening tool for detecting breast changes. They were also willing to seek professional help in case of finding any breast changes while performing BSE.

This optimistic belief in the examination and the willingness to seek help warrant positive steps to be taken toward popularizing BSE. India already has an Anganwadi system, wherein women and children are cared for and educated about health topics at the grassroots level. Training the Anganwadi workers can be helpful in quickly teaching the masses about the practices of BSE and disseminating correct information regarding breast cancer to curb the mortality and morbidity associated with it. The study also highlighted the untapped potential of information dissemination through teachers and family elders. School teachers could be trained to inform young girls about the benefits of practicing BSE and help raise a healthier and more informed population.

This study included a large sample of women from various economic and educational strata of society. Since the data were collected from only one center, the findings may not be extrapolated and generalized to the rest of the country. The study also did not collect any information about variability in knowledge with the age of participants. The age dependence of knowledge, if any, could have helped policymakers recognize the target age group for intervention and education for breast cancer prevention.

## Conclusions

BSE is a misunderstood and underused tool for managing breast health. The lack of awareness among women about the practices of BSE stems from the paucity of information sources. Although mass media plays a role in disseminating information, it alone is not enough. This study highlighted the need to educate women in depth about the steps, practices, and frequency of performing BSE. Hospitals, health care workers, and educational institutes have much untapped potential to disseminate information. However, before we embark on the mission, we need to equip and empower educators with the correct information, starting at the level of health care workers, social workers, "Anganwadi" workers, and even school teachers, to ensure more comprehensive coverage of the population. Doctors, nurses, and other medical staff need to be trained heavily to answer any doubts of the aforementioned mass educators. Women must be taught about the danger signs of breast malignancy and when to seek professional help. This will aid in the judicious use of scarce resources to allocate preferred services of CBE and radiological imaging such as mammograms to women with danger signs. Early detection of malignant changes will promote timely intervention and reduce breast cancer-related deaths.

## Appendices

Breast Cancer and Breast Self-Examination Questionnaire

1.  Do you consent to provide basic information about your knowledge of breast self-examination? Yes/No

2.  Age (years):

3.  Marital status: Single/Never married/Married/Divorced/Widowed/Cohabilitating/Separated

4.  Religion:

5.  Occupation: Doctor/Medical student/Nurse/Other hospital staff/None of these

6.  Diet: Veg/Non-veg

7.  Have you heard about Breast Cancer? Yes/No

8.  What are your source(s) of information? Books/Media (TV, Radio, Internet, etc.)/Health care industry/Lecture/Conferences/Seminars

9.  Has any member of your family been diagnosed with breast cancer? Yes/No

10.  Which of these are risk factors of breast cancer?

Obesity/Alcohol/Smoking

Increasing age/Family history

Use of oral contraceptives

Poor sleep/Heavy lifting

Don't know

11.  Which of these are signs of breast cancer?

Lump in the breast or armpit/Discharge from nipple or retraction of nipple/Skin changes on breast/Loss of weight/Difficulty breathing

12.  Have you heard of breast self-examination? Yes/No

13.  Have you been taught how to do BSE? Yes/No

14.  Who taught you? Parents or Relative/Teacher/Doctor or Nurse/Friend/Other

15.  At what age should BSE be started? From birth/From 20 years/From 30 years/After menopause/No idea

16.  How often should BSE be done? Daily/Weekly/Monthly/Yearly/No idea

17.  What is the best time to do BSE? During menstrual flow/A week after period/During pregnancy/During breast feeding/No idea

18.  BSE is done by: Feeling the breast or armpit with the hand/Doing ultrasound of the breast/Mammography/Inspecting the breast in the mirror

19.  If you discover any abnormality during BSE, what will you do? Pray over it/Do some lab tests/See a doctor/Do nothing

20.  What are the benefits of BSE? Early detection of breast cancer/Detection of any abnormal changes in the breast/A good breast exercise/No benefits

21.  Do you practice BSE? Yes/No

22.  How often do you practice BSE? Weekly/Monthly/Occasionally/Rarely

23.  If you have been practicing BSE, have you ever discovered any abnormalities in your breast? Yes/No/I have not done BSE before

24.  Do you think BSE is a good practice? Yes/No
